# Acellular Pertussis Vaccines Induce Anti-pertactin Bactericidal Antibodies Which Drives the Emergence of Pertactin-Negative Strains

**DOI:** 10.3389/fmicb.2020.02108

**Published:** 2020-08-27

**Authors:** Elodie Lesne, Breeze E. Cavell, Irene Freire-Martin, Ruby Persaud, Frances Alexander, Stephen Taylor, Mary Matheson, Cécile A. C. M. van Els, Andrew Gorringe

**Affiliations:** ^1^Public Health England, Porton Down, United Kingdom; ^2^Centre for Infectious Disease Control, National Institute for Public Health and the Environment, Bilthoven, Netherlands

**Keywords:** *Bordetella pertussis*, complement, antigens, antibodies, bactericidal activity, vaccine, pertactin

## Abstract

Despite high vaccination coverage, *Bordetella pertussis* the causative agent of whooping cough is still a health concern worldwide. A resurgence of pertussis cases has been reported, particularly in countries using acellular vaccines with waning immunity and pathogen adaptation thought to be responsible. A better understanding of protective immune responses is needed for the development of improved vaccines. In our study, *B. pertussis* strain B1917 variants presenting a single gene deletion were generated to analyze the role of vaccine components or candidate vaccine antigens as targets for bactericidal antibodies generated after acellular vaccination or natural infection. Our results show that acellular vaccination generates bactericidal antibodies that are only directed against pertactin. Serum bactericidal assay performed with convalescent samples show that disease induces bactericidal antibodies against Prn but against other antigen(s) as well. Four candidate vaccine antigens (CyaA, Vag8, BrkA, and TcfA) have been studied but were not targets for complement-mediated bactericidal antibodies after natural infection. We confirm that Vag8 and BrkA are involved in complement resistance and would be targeted by blocking antibodies. Our study suggests that the emergence and the widespread circulation of Prn-deficient strains is driven by acellular vaccination and the generation of bactericidal antibodies targeting Prn.

## Introduction

*Bordetella pertussis*, the causative agent of whooping cough, is a gram-negative bacterium that colonizes the human respiratory tract. Despite high vaccination coverage, this pathogen is highly circulating in a range of populations (Barkoff et al., 2015) and induces more than 24.1 million cases and 160 700 deaths per year worldwide in children younger than 5 years (Yeung et al., 2017).

In the 1940s, inactivated whole-cell (wP) *B. pertussis* vaccines were introduced and considerably reduced the incidence of the disease. wP vaccines were then replaced in many countries by less reactogenic acellular (aP) vaccines, composed of one to five purified and detoxified antigens from the bacterium (pertussis toxin, filamentous hemagglutinin, pertactin and fimbriae 2 and 3) (Gustafsson et al., 1996). In the last two decades, the incidence of pertussis has been increasing in several countries which have high aP vaccine coverage (Nieves and Heininger, 2016; Pinto and Merkel, 2017; Esposito et al., 2019). This is likely to be caused by a number of factors including a shorter duration of protection provided by aP than wP vaccines (Witt et al., 2013; Klein et al., 2016), a greater circulation of *B. pertussis* in aP-vaccinated populations (Althouse and Scarpino, 2015) and evolution of strains with greater fitness (Belcher and Preston, 2015). Mechanisms involved in pathogen adaptation include allelic and antigenic variations, emergence of strains with increased pertussis toxin production or strains deficient in Prn, PTX or FHA (Bart et al., 2014a; Bouchez et al., 2015; Xu et al., 2015; Williams et al., 2016; Weigand et al., 2018; Barkoff et al., 2019). Pertactin-deficient strains are by far the most commonly reported and in some countries using aP vaccination, the prevalence has reached around 80% (Lam et al., 2014; Martin et al., 2015).

Protection against *B. pertussis* requires humoral and cellular immune responses (Higgs et al., 2012), with strong IgG responses induced after disease and vaccination. Natural infection or wP vaccination induce Th1/Th17-dominated responses involved in long lasting immunity, while aP vaccination induces Th2-dominated responses (Ross et al., 2013; da Silva Antunes et al., 2018). Cellular and humoral polarizations are determined by primary vaccination and cannot be reprogrammed after boosting (van der Lee et al., 2018). Several studies performed in animal models have shown that aP vaccination protects against lung infection but failed to prevent nasal colonization and transmission, whereas wP vaccination reduces colonization and transmission (Warfel et al., 2014; Wilk et al., 2019).

One of the first lines of defense against microorganisms is the human complement system. It is composed of more than 30 proteins expressed in the blood and on mucosal surfaces of the respiratory tract, and can be activated by three pathways, the classical, the lectin and the alternative pathways. After activation by one of these pathways, a cascade of enzymatic reactions allows the cleavage of C3 by C3 convertases and the release of C3b involved in the opsonization of the bacteria. C3b deposition on bacteria followed by the formation of C5 convertase and the cleavage of C5 can also lead to the formation of the membrane attack complex and the lysis of gram-negative bacteria (Jongerius et al., 2015).

Several studies have reported that *B. pertussis* has developed strategies for immune evasion, including the ability to bind complement-regulatory proteins (Barnes and Weiss, 2001; Berggård et al., 2001; Amdahl et al., 2011; Marr et al., 2011; Geurtsen et al., 2014; Hovingh et al., 2017). The two Bvg-activated autotransporter proteins Vag8 and BrkA are known to be involved in complement resistance by binding C1 esterase inhibitor (C1-INH) (Marr et al., 2011) or preventing the deposition of C3 onto the bacteria and the formation of the membrane attack complex (Barnes and Weiss, 2001). *B. pertussis* is also able to bind C4BP, a plasma protein that inhibits the classical pathway via FHA and at least one other Bvg-regulated gene (Berggård et al., 2001). It has been observed that recently-isolated strains vary in their interaction with human complement components such as C3b/iC3b, C5b-9 and C1-INH with some isolates showing greater resistance to killing in serum (Brookes et al., 2018).

To colonize the human respiratory tract, *B. pertussis* produces several virulence factors including adhesins and toxins (Locht et al., 2001). Previous studies have analyzed the role of key antigens *in vivo* and shown that some toxin (PTX and CyaA), adhesin (FHA and Fim) and autotransporter (TcfA and BrkA) deficient mutants exhibit a defective colonization of the mouse respiratory tract (Mooi et al., 1992; Fernandez and Weiss, 1994; Finn and Stevens, 1995; Carbonetti et al., 2004, 2005). The two autotransporters, Prn and Vag8, were reported to induce protection against *B. pertussis* lung infection following intranasal challenge (Roberts et al., 1992; De Gouw et al., 2014).

Evaluation of several bacterial and viral vaccines has shown that the prevention of infection correlates with the induction of specific antibodies which can be opsonophagocytic, bactericidal or binding antibodies (Plotkin, 2020). So far, no clear correlate of protection against *B. pertussis* has been determined. Evidence from household contact studies linked to aP vaccine trials suggested that pre-exposure levels of anti-Prn and anti-fimbriae IgG correlated with protection against typical and mild pertussis, where as anti-PTX IgG only correlated with protection against typical pertussis (Olin et al., 2001). It appears that natural infection triggers a more efficient and long-lasting immunity than aP vaccination, suggesting that other antigens than the five already included in the vaccine composition may improve efficacy. A better understanding of the function of the antibodies associated with specific antigens will help in the development of new vaccines and in the establishment of correlate(s) of protection for pertussis.

In this study, *B. pertussis* strain B1917 variants with a single gene deletion were generated and used with human complement to analyze the role of vaccine components or candidate vaccine antigens and to study the bactericidal activity of the antibodies generated after acellular vaccination or natural infection. aP vaccination generates bactericidal antibodies directed only against Prn but other antigens in addition to Prn generate bactericidal antibodies after disease.

## Materials and Methods

### Bacterial Growth Conditions

*Bordetella pertussis* B1917 (Bart et al., 2014b) and derivative strains were cultured on blood charcoal agar plates (Oxoid) for 2 days at 35°C prior to seeding into THIJS medium (Thalen et al., 1999) supplemented with 0.75 mM Heptakis-(2,6-di-O-methyl)-β-cyclodextrin (Daito Pharmaceutical Company) at OD_600__nm_ 0.1 and cultured for 16 h at 35°C with orbital shaking. Culture medium was supplemented with 50 μg/mL of kanamycin for bacteria carrying the modified pBBR plasmid. Then, mid-exponential phase bacteria were harvested and stocks were made in THIJS medium with 10% glycerol and stored at −80°C. Prior to each assay, bacteria were warmed at 37°C for 30 min.

### Bacterial Strains: Generation of Knock-Out Mutants and Pertactin Complemented Strain in B1917

Generation of knock-out strains was based on the method already described (Brookes et al., 2018). Briefly, 5’ and 3’ flanking regions of targeted genes were amplified by PCR using primers containing *Bsa*I sites (Supplementary Table S1) to be introduced by Golden Gate assembly into the intermediate pCR8Gw plasmid. The assembled regions were then transferred into a suicide vector pSS4940GW using gateway cloning. To construct the pSS4940GW-Δ*cyaA*, the flanking regions of *cyaA* were amplified by PCR using CyaB and CyaC primers (Supplementary Table S1), following by overlapping PCR (over-CyaB-Rv and over-CyaC-Fw primers, Supplementary Table S1). The amplicon was then introduced in the final vector using *Eco*RI-*Xho*I restriction enzymes. Conjugation between the donor strain *E. coli* ST18 and the receiver strain B1917 allowed the deletion of the targeted genes in the *B. pertussis* chromosome by homologous recombination.

The entire gene of Prn and its 5’ region was amplified by PCR using primers containing *Spe*I and *Hin*dIII restriction sites (Supplementary Table S1) and introduced into the modified pCR8Gw plasmid. For the latter, *Spe*I and *Hin*dIII restrictions sites were introduced by site-directed mutagenesis using respective primers (Supplementary Table S1). The assembled regions were transferred into a replicative vector pBBRKanR-Gw using gateway cloning before insertion into the deleted strain by conjugation with the *E. coli* ST18 strain.

### Vaccination and Convalescent Study Population and Sample Collection

Pre- and post-aP vaccination sera were obtained from the MULTIBOOST study (NCT02526394) in which United Kingdom teenagers (13.5–17 years of age) were vaccinated with a pertussis booster dose of either IPVBoostrix or Repevax. Two blood samples were collected one prior to vaccination and 35 weeks later. All participants provided written informed consent. For participants under 16, written informed consent was provided by parents or guardians of participants. The MULTIBOOST study was approved by the MHRA and the NRES Committee London – Brent, REC reference 13/LO/0681. The Eudract registration was 2012-005273-31.

Convalescent individuals were cases of all ages with a recent laboratory-proven clinical symptomatic *B. pertussis* infection from two Dutch observational studies, SKI (NL16334.040.07) and Immfact (NL4679.094.13), both approved by the accredited Review Board METC UMC Utrecht. All participants provided written informed consent. For minor participants written informed consent was provided by both parents or guardians of participants. These studies were conducted in compliance with the principles of the Declaration of Helsinki. Plasma samples from sixty eight cases (age 0,4–76,5 years, median 15,3 years; male/female ratio 0,47/0,53), collected 2–15 weeks (average 4.8 ± 1.7 weeks) post-diagnosis, were isolated and stored for serological analysis as described earlier (Hovingh et al., 2018).

### *Bordetella pertussis* Specific Antibody Concentrations of Clinical Samples

PTX, FHA, Prn, and Fim2/3 antibody concentrations in IU/mL were previously determined by ELISA using the method published in Ladhani et al. (2015) for the MULTIBOOST sera or by multiplex immunoassay as published in Van Twillert et al. (2017) for the convalescent plasma samples.

### Human Complement Source and Complement Resistance Assay

IgG- and IgM-depleted human plasma used as complement source was prepared as described by Brookes et al. (2013) with IgM removed using a Poros Capture Select column (Thermofisher) attached to an AKTA purifier (GE). The complement component activity and functional activity were determined as described in Brookes et al. (2013).

Sensitivity of the B1917 variants to complement alone was determined by incubating 10 μl of 8 × 10^4^ CFU/mL bacteria in 20 μl HBSS and 0.5% BSA with 10 μl of active or heat-inactivated IgG- and IgM-depleted human plasma to obtain a final concentration of 10%. The heat inactivation of the complement source was performed prior to the assay at 56°C for 30 min. After 2 h at 37°C with shaking at 900 rpm, 10 μl of bacteria were plated out onto a blood charcoal agar plate using the tilt method and CFU were counted after five days at 35°C.

### Serum Bactericidal Assay (SBA)

Prior to the assay, heat inactivation of the sera or plasma was performed at 56°C for 30 min. Twofold serial dilutions of heat-inactivated samples with HBSS and 0.5% BSA were performed in 20 μl final volume in a microplate. Bacteria from a frozen mid-exponential phase THIJS medium liquid culture were warmed at 37°C for 30 min before being diluted in the buffer to 8 × 10^4^ CFU/mL and 10 μl added in each well. 10 μl of IgG- and IgM-depleted human plasma was added to obtain a final concentration of 10%. The plate was then incubated for 2 h at 37°C with shaking at 900 rpm. 10 μl from each well was plated out onto a blood charcoal agar plate using the tilt method and then incubated for five days at 35°C. Colonies were counted and interpolated reciprocal titers were assigned as the serum dilution that gives 50% survival compared to the complement-only CFU count. Heat-inactivated 1st WHO International Standard pertussis antiserum (NIBSC 06/140) was included in every plate as a control. A value of 4 or 32768 was arbitrarily reported when no titer could be assigned using the first (1:8) or last (1:16384) dilution, respectively.

### Bacterial IgG Binding Assay

Prior to the assay, heat inactivation of clinical serum or plasma samples was performed at 56°C for 30 min. 2 μl of heat-inactivated sample was incubated in duplicate with 198 μl of bacteria at an OD_600__nm_ of 0.1 in PBS with 2% BSA for 30 min at 25°C and 900 rpm. Heat-inactivated 1st WHO International Standard pertussis antiserum (NIBSC 06/140) was tested as a control. The samples were centrifuged at 3060 × *g* for 5 min and washed with PBS. Pellets were then resuspended in 200 μl anti-mouse or anti-human IgG -FITC (Jackson Immunochemicals) at 1:500 and incubated for 20 min in the dark at room temperature. A washing step was then performed, and the pellets were resuspended in 200 μl PBS with 2% formaldehyde. After 1-hour incubation, a washing step was performed, and bacteria were labeled with 100 μl Live/Dead Violet stain at 1:500 (Invitrogen) for 30 min at room temperature in the dark. Following a further washing step, pellets were resuspended in 200 μl PBS and analyzed on a Flow Cytometer (CytoFLEX S, Beckman, Coulter, United Kingdom). Median fluorescence intensities were calculated for each individual or control sample using CytExpert software. The median background fluorescence intensity was removed from each sample value.

### Statistics

Statistical analyses were performed using GraphPad Prism 8 software. Distribution of the data was assessed before performing one-way ANOVA followed by a multi-comparison test to compare more than two strains to the WT strain and Student *t*-test to compare the complemented Prn strain to the WT strain. Paired statistical tests were used to analyze data obtained with pre-aP, post-aP and convalescent samples across strains. Pearson correlation test was performed on log10 transformed values.

## Results

### Generation of Knock-Out Variants Deficient in Each of the Acellular Vaccine Antigens

**FIGURE 1:**
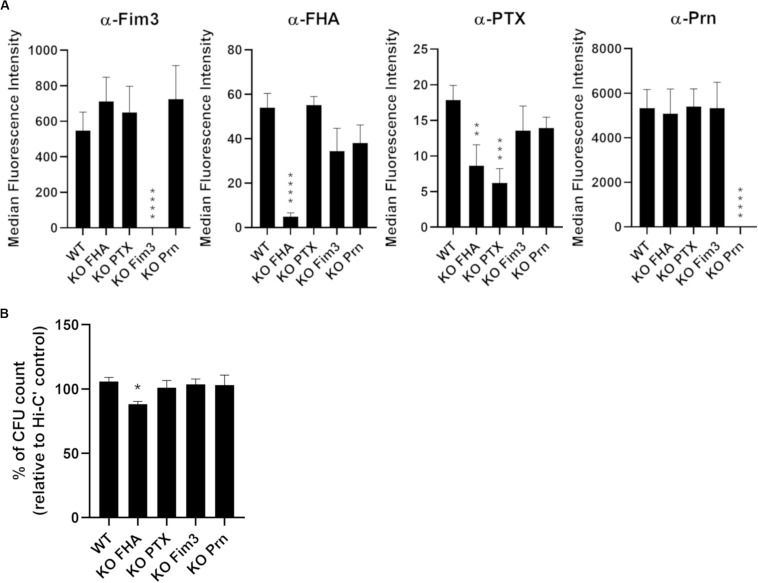
Characterization of knock-out variants deficient in each of the acellular vaccine antigens. **(A)** Quantification of IgG binding against Fim3, FHA, PTX, and Prn onto B1917 (WT) and KO variants. The fluorescence intensity was determined from two separate experiments with at least technical duplicates. Means and standard deviation errors are represented. Statistical analyses were performed, and significant *p* values relative to WT strain are indicated (****p ≤ 0.0001, ***p ≤ 0.001, **p ≤ 0.01). **(B)** Sensitivity of B1917 (WT) and KO variants to complement alone was assessed. The values correspond to the CFU count from bacteria incubated with active complement relative to the CFU count from bacteria incubated with heat-inactivated complement (Hi-C’). The experiment including technical replicates was repeated at least three times and means and standard deviation errors are represented. Statistical analyses were performed, and significant *p* values relative to WT strain are indicated (*p ≤ 0.05).

To determine the importance of *B. pertussis* aP vaccine antigens for antibody and complement-dependent killing of *B. pertussis*, single deletions of the entire gene of *fhaB*, *ptxABDEC*, *fim3*, and *prn* were performed in the B1917 background by homologous recombination. Complete gene deletions were checked by PCR followed by Sanger sequencing analyses. The phenotype of each of the B1917 variants was then analyzed by measuring the surface binding of monoclonal antibodies directed against either Fim3, FHA, PTX or Prn. Quantification of IgG binding onto the strains was assessed using a fluorescent secondary antibody by flow cytometry (Figure 1A). As expected, specific IgG binding is reduced for the respective variants compared to the WT strain, confirming the deletion of the targeted antigens. The low fluorescence intensity values obtained with the anti-FHA and anti-PTX monoclonal antibodies (Figure 1A, middle panels) can be explained by the low amount of FHA and PTX expressed at the surface of washed bacteria as these antigens are mainly secreted. In addition, FHA may act to stabilize PTX at the bacterial surface.

Prior to performing serum bactericidal assays (SBA), the sensitivity of the B1917 variants to complement alone was checked. The bacteria were incubated for 2 h with 10% IgG- and IgM-depleted human plasma as the complement source and CFU counted (Figure 1B). No sensitivity to complement alone was observed for B1917 and KO PTX, KO Fim3 and KO Prn variants. B1917 KO FHA presents a slightly increased sensitivity to complement alone (around 10% reduction of CFU count).

### Booster aP Vaccination Induces Bactericidal Antibodies Only Against Prn

**FIGURE 2:**
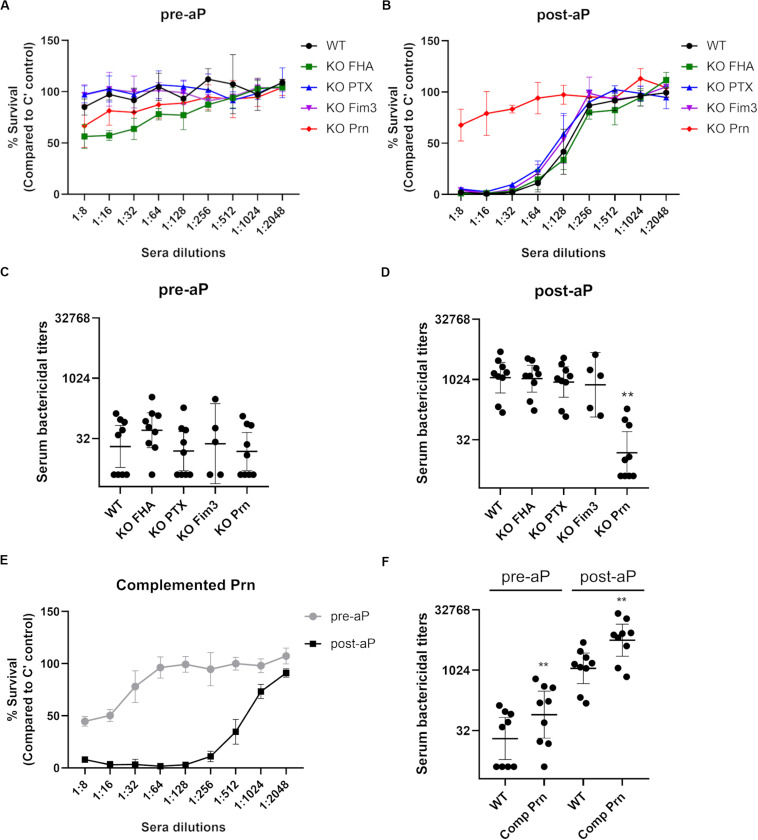
Serum bactericidal activity of pre- and post-aP vaccine samples with B1917 (WT) and KO variants. **(A,B)** Survival curves with a pre-aP **(A)** or post-aP **(B)** convenience serum from the same individual represent the percent of CFU count from bacteria incubated with serial dilutions of serum relative to bacteria incubated with complement (C’) only. The bars represent the mean of at least three separate experiments and standard deviation error is given. **(C,D)** Serum bactericidal titers were assigned as the interpolated serum dilution which gives 50% of bacterial killing when incubating the bacteria with serial dilutions of pre-aP **(C)** or post-aP **(D)** vaccine serum and active complement. 9 pre- and post-aP vaccine sera were analyzed and only sera from individuals who had received a Fim-containing vaccine (Repevax, *n* = 5) were tested for KO Fim3. The assays were performed at least three separate times and geometric means with 95% confidence interval are represented. Statistical analyses were performed for each data point using the corresponding WT data point as a control, and significant *p* values are indicated (**p ≤ 0.01). **(E)** Survival curves of the complemented Prn strain with matched pre- and post-aP sera. The bars represent the mean of at least three separate experiments and standard deviation error is given. **(F)** Serum bactericidal titers obtained for the complemented Prn (Comp Prn) strain incubated with 9 pre- and post-aP sera. The assays were performed at least three separate times and geometric means with 95% confidence interval are represented. Statistical analyses were performed for each data point using the corresponding WT data point as a control, and significant *p* values are indicated (**p ≤ 0.01).

Convenience samples of pre- and post-vaccine sera from United Kingdom teenagers were used in a serum bactericidal assay (SBA) to analyze the function of the antibodies generated after acellular booster (five individuals received the Fim-containing vaccine, Repevax, and four received IPVBoostrix, Figure 2). After aP vaccination, the level of IgG directed against the antigens included in the vaccine formulation was increased for each individual (data not shown). With dilutions of pre-vaccination sera, all the strains show similar survival curves and a representative result is shown in Figure 2A. Killing of the WT and KO strains was seen at greater serum dilutions of post-aP vaccination sera, except for the KO Prn variant (Figure 2B). The interpolated dilutions of serum giving 50% or greater bacterial killing for 9 pre- (Figure 2C) and post-aP (Figure 2D) vaccination sera show similar SBA titers with all variants except for KO Prn. Approximately 32-fold greater SBA titers with post-aP vaccination sera were observed for WT and all the KO variants. In contrast, similar SBA titers are obtained for KO Prn with pre- and post-aP vaccination sera. This suggests that it is only the anti-Prn antibodies generated after the acellular booster that are bactericidal. Surface antibody binding assays were performed, and results are reported relative to those of B1917 (Supplementary Figures S1A,B). Only total IgG binding onto the B1917 KO Prn is significantly lower than that obtained with the WT strain. The KO Fim3 strain presents a slightly lower total IgG binding compared to the WT strain, but this difference is not significant. No difference in total IgG binding is observed with KO FHA and KO PTX probably due to the low expression of these antigens on the surface of the WT strain (Figure 1A).

To confirm that the generation of the KO strains does not induce a genetic rearrangement responsible for the low SBA titers obtained for the KO Prn strain after vaccination, a pertactin complemented strain was generated by introducing the Prn promotor and its complete gene in the KO Prn variant using a low copy replicative plasmid. This strain expresses a higher level of Prn than the WT strain as highlighted by total IgG binding using an anti-Prn monoclonal antibody (Supplementary Figure S1C), likely due to the presence of more than one plasmid expressing Prn per bacteria. No change in sensitivity to complement alone was determined (data not shown). The Prn complemented strain gave increased SBA titers with post vaccination sera compared to pre-vaccine sera (Figures 2E,F), confirming that anti-Prn antibodies generated after acellular booster are bactericidal. The slightly increased SBA titers compared to B1917 are explained by a higher total IgG binding onto the complemented strain (Supplementary Figures S1C,D).

**FIGURE 3:**
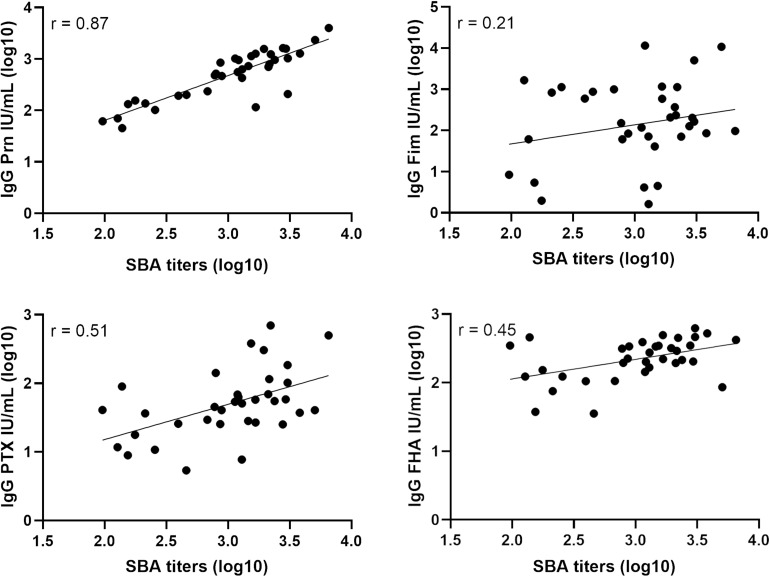
Correlation of serum bactericidal titers and IgG levels for post-aP vaccine sera. Serum bactericidal titers obtained with the WT strain were compared to the IgG level of anti-Prn, anti-Fim, anti-PTX and anti-FHA from 35 post-aP vaccine sera of which 16 received Repevax (Fim-containing vaccine) and 19 IPVBoostrix.

The anti-Prn, anti-PTX, anti-FHA and anti-Fim IgG concentrations of the post-aP vaccination sera were determined by ELISA for 35 samples in total. 16 sera were from individuals who had received a Fim-containing vaccine (Repevax) and the remaining 19 sera were from individuals who were administered IPVBoostrix. A high correlation (0.87) is obtained when comparing the post-aP SBA titers with B1917 and the anti-Prn IgG concentrations in post-aP vaccine sera (Figure 3). Weaker correlations of 0.51 and 0.45 are observed for anti-PTX and anti-FHA, respectively. A poor correlation is obtained for anti-Fim (0.21).

### Disease Induces Bactericidal Antibodies Against Prn and Other *B. pertussis* Antigens

**FIGURE 4:**
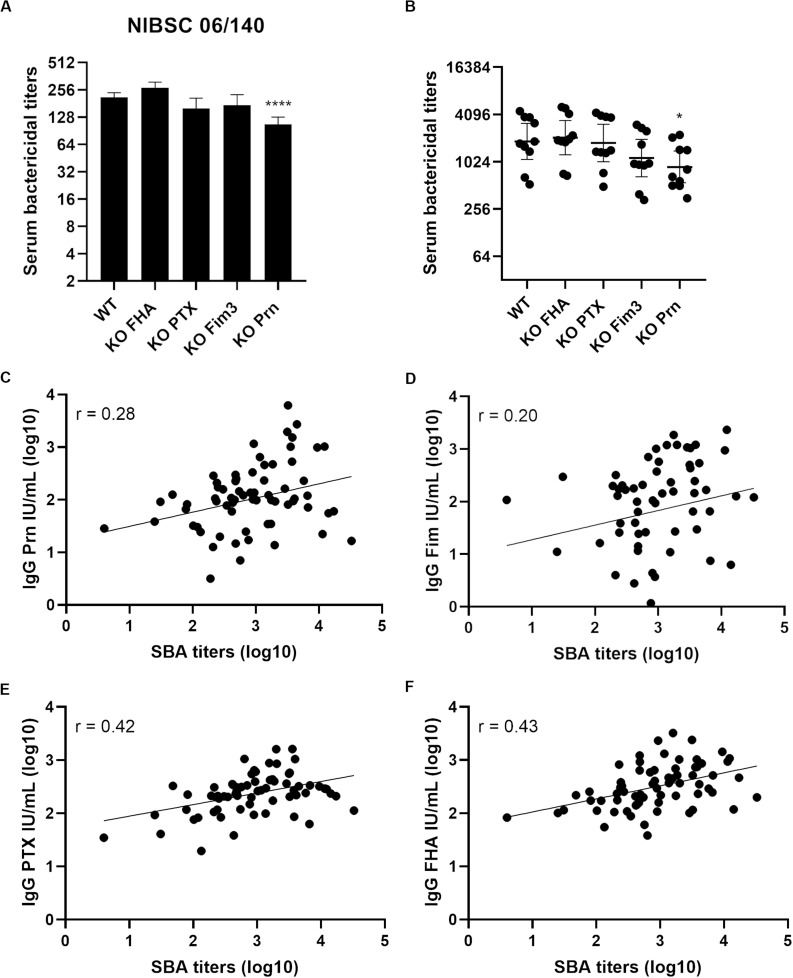
Serum bactericidal activity of convalescent samples with B1917 (WT) and KO variants deficient in each of the acellular vaccine antigens. **(A,B)** SBAs were performed using the 1st WHO International Standard pertussis antiserum NIBSC 06/140 **(A)** and 10 convalescent **(B)** samples. Serum bactericidal titers were assigned as the interpolated serum dilution which gives 50% of bacterial killing when incubating the bacteria with serum and active complement. The assays were performed at least three separate times for panel **(A)** and at least once for panel **(B)** and geometric means with 95% confidence interval are represented. Statistical analyses were performed for each data point using the corresponding WT data point as a control, and significant *p* values are indicated (****p ≤ 0.0001, *p ≤ 0.05). **(C–F)** Serum bactericidal titers obtained with the WT strain were compared using Pearson’s correlation to the IgG level of anti-Prn **(C)**, anti-Fim **(D)**, anti-PTX **(E)** and anti-FHA **(F)** from 68 convalescent individuals of different ages who received pertussis vaccination as a child.

Serum bactericidal assays with WT and aP vaccine antigen KO strains were performed using the 1st WHO International Standard pertussis antiserum (Figure 4A) which is a pool from convalescent individuals. No significant difference was observed when comparing the SBA titers with this serum obtained with KO FHA, KO PTX, and KO Fim3 variants to those obtained with the WT strain (Figure 4A). A slightly lower SBA titer was observed with KO Prn variant consistent with lower total IgG binding onto its bacterial surface (Supplementary Figure S2A).

A convenience set of ten convalescent samples from young Dutch children under 15 was used to determine the bactericidal activity of the antibodies generated following natural infection (Figure 4B). The KO FHA, KO PTX, and KO Fim3 variants show similar SBA titers to WT, suggesting that the remaining antigens generate the complement-mediated bactericidal antibodies seen following disease. The SBA titers obtained with the KO Prn variant were slightly lower than those obtained for the WT, highlighting that anti-Prn antibodies are a component of the antibodies generated following natural infection which are responsible for antibody and complement-mediated killing observed. Consistent with this, the KO Prn variant presented a significantly lower total IgG binding onto its surface compared to the WT strain (Supplementary Figure S2B).

The anti-Prn, anti-PTX, anti-FHA and anti-Fim IgG concentrations were determined by multiplex immunoassay for samples from 68 convalescent individuals of different ages who received pertussis vaccination as a child. A poor correlation is obtained when comparing the SBA titers of B1917 and all the anti-pertussis IgG measured (0.20 for anti-Fim, 0.28 for anti-Prn, 0.42 for anti-PTX and 0.43 for anti-FHA IgG levels in convalescent samples, Figures 4C–F).

### Serum Bactericidal Activity Against Candidate Vaccine Antigens

**FIGURE 5:**
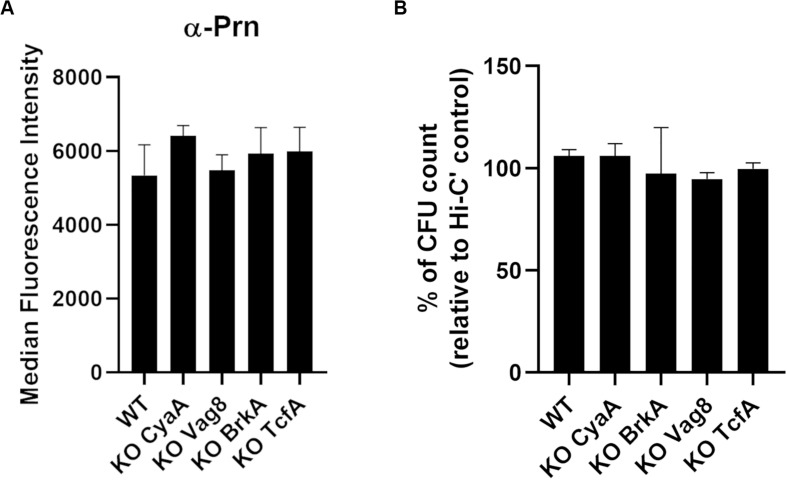
Characterization of knock-out variants deficient in vaccine candidate antigens. **(A)** Quantification of IgG binding against Prn onto B1917 (WT) and KO variants. The fluorescence intensity was determined from two separate experiments with technical duplicates and means and standard deviation errors are represented. Statistical analyses were performed, and no significant *p* value relative to WT strain was observed. **(B)** Sensitivity of B1917 (WT) and KO variants to complement alone was assessed. The values correspond to the CFU count from bacteria incubated with active complement relative to the CFU count from bacteria incubated with heat-inactivated complement (Hi-C’). The experiment including technical replicates was repeated at least three times and means and standard deviation errors are represented. Statistical analyses were performed, and no significant *p* value relative to WT strain was determined.

In addition to aP antigens, we focused on several antigens that have been reported to be potential candidates for aP vaccination (Marr et al., 2008; De Gouw et al., 2014; Sebo et al., 2014; Jongerius et al., 2015; Luu et al., 2020). Single deletion of *cyaA*, *vag8*, *brkA*, and *tcfA* genes was performed in the B1917 background by homologous recombination. Gene deletions were checked by PCR followed by Sanger sequencing analyses. For each variant, the expression of Prn was determined by antibody binding, and no significant difference of expression was observed (Figure 5A).

Complement sensitivity of the B1917 variants was assessed by incubating the bacteria for 2 h with 10% IgG- and IgM-depleted human plasma as a complement source (Figure 5B). No difference in sensitivity to complement alone was observed for B1917 and KO CyaA, KO Vag8, KO BrkA, and KO TcfA variants.

**FIGURE 6:**
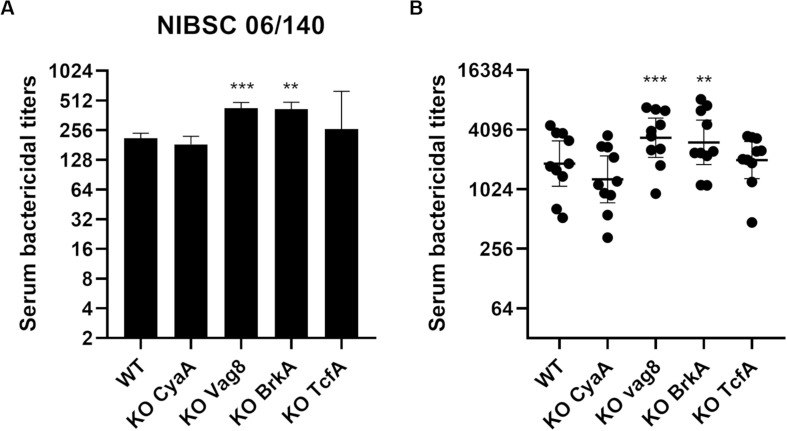
Serum bactericidal activity of convalescent samples with B1917 (WT) and KO variants deficient of vaccine candidate antigens. **(A,B)** SBAs were performed using the 1st WHO International Standard pertussis antiserum NIBSC 06/140 **(A)** and 10 convalescent **(B)** samples. Serum bactericidal titers were assigned as the interpolated serum dilution which gives 50% of bacterial killing when incubating the bacteria with serum and active complement. The assays were performed at least three separate times for panel **(A)** and at least once for panel **(B)** and the geometric means with 95% confidence interval are represented. Statistical analyses were performed for each data point using the corresponding WT data point as a control, and significant *p* values are indicated (***p ≤ 0.001, **p ≤ 0.01).

Serum bactericidal assays were performed using the 1st WHO International Standard pertussis antiserum (Figure 6A). KO Vag8 and KO BrkA present higher serum bactericidal titers than the WT strain, suggesting that these two antigens are involved in resistance to antibody and complement-mediated killing. Both KO Vag8 and KO BrkA present a similar level of total IgG binding onto its surface compared to the WT strain, suggesting that the increase of SBA titers is not a consequence of an increase of bactericidal IgG binding to the surface of the bacteria (Supplementary Figure S2A). No significant difference in SBA titers was observed with KO CyaA and KO TcfA compared to WT (Figure 6A). These two variants have a slightly higher total IgG binding onto their surface with NIBSC 06/140 than for WT. This increase could be due to a better accessibility of the surface antigens as the other antigens are not expressed in the variants. Despite these two variants binding more IgG, similar SBA titers to WT were observed (Figure 6A).

The convenience set of ten convalescent samples from children under 15 were also assessed in SBA with the B1917 variants (Figure 6B). Again, there is an increase in SBA titers with KO Vag8 and KO BrkA variants compared to B1917, with no significant difference in total IgG binding compared to the WT (Supplementary Figure S2B). The absence of these two antigens appears to cause greater antibody and complement-mediated killing. Similar SBA titers to WT were observed with KO CyaA and KO TcfA. The latter shows slightly higher total IgG binding onto its surface compared to the WT (Supplementary Figure S2B). The results suggest that antibodies directed against other antigens are responsible for the complement-mediated bactericidal activity observed after natural infection in the absence of these antigens on the target strain. To ensure that the results obtained with the convalescent samples are not due to a combined effect between the presence of bactericidal antibodies and a complement resistance ability of the strains, SBA with seven post-aP vaccine serum was performed. Similar results were obtained (Supplementary Figure S3) with a slight increase of SBA titers observed with KO Vag8 and KO BrkA, although no significant difference with WT was determined. Similar SBA titers to B1917 are observed for KO CyaA and KO TcfA, suggesting that the latter are not involved in complement resistance.

## Discussion

Despite high vaccination coverage, a resurgence of pertussis cases has been reported, particularly in countries using aP vaccines. It has been suggested that aP vaccines protect from the disease but fail to prevent colonization, leading to asymptomatic carriers who can spread the disease to susceptible people, including non or partly immunized children or individuals whose immunity has waned. To develop improved pertussis vaccines, an enhanced knowledge of immune correlates of protection is required (Diavatopoulos et al., 2019). Bactericidal antibodies have been shown to correlate with protection for several bacteria including *Haemophilus influenzae* type B and *Neisseria meningitidis* vaccines (Fothergill and Wright, 1933; Goldschneider et al., 1969). Antibody and complement are present on the mucosal surface (Persson et al., 1991; Lamm, 1997; Jongerius et al., 2015) and bactericidal antibodies may play a role in protection from pertussis infection. Thus, we have studied complement-mediated bactericidal activity of post-aP vaccine and convalescent sera against WT and KO variants lacking aP vaccine antigens and several candidate vaccine antigens to determine their role as targets for bactericidal antibodies.

In the pertussis field, the complement-mediated bactericidal activity of antibodies has been assessed by a radial diffusion serum assay (Weiss et al., 1999; Oliver and Fernandez, 2001) or a bactericidal assay using as complement source either guinea pig serum (Weingart et al., 2000a; Riaz et al., 2015), precolostral calf serum (Gotto et al., 1993; Kubler-Kielb et al., 2011) or endogenous complement present in the sample (Weiss et al., 1999). Studies performed on *N. meningitidis* suggest that the source of complement is important to accurately quantify serostatus and human complement is the preferred source of complement to use for serogroup B *N. meningitidis* assays (Santos et al., 2001; Gill et al., 2011; Findlow et al., 2019). Various concentrations of complement have been used and differences in growth conditions have been reported. In our study, bacterial culture was standardized using the defined THIJS medium and harvested in the middle log-phase for each variant. It has been reported that *B. pertussis* complement susceptibility is growth stage-specific. Bacteria in stationary phase were more resistant than bacteria in logarithmic phase (Barnes and Weiss, 2002). Moreover, in THIJS medium, the exponential phase is the optimal harvest point for the cultivation of *B. pertussis* for vaccine, as during this phase there is a higher expression of the virulence factors (Van De Waterbeemd et al., 2009). To remove the effect of endogenous complement, the test sera were heat-inactivated and an external complement source used. The use of IgG- and IgM-depleted human plasma as the complement source ensures that only the bactericidal activity of the test sera is measured. Results obtained from serum bactericidal assays only reflect a portion of the bactericidal activity of antibodies in blood or on mucosal surfaces as immune effector cells and other innate mechanisms such as antimicrobial peptides will contribute to killing of infecting *B. pertussis in vivo* (de Gouw et al., 2011).

Our study shows that the autotransporter Prn is not involved in resistance to complement-mediated killing but is able to generate bactericidal antibodies after aP vaccination or natural immunization. These results are consistent with previous studies that have shown Prn is a protective antigen, reducing respiratory tract colonization of mice after immunization and eliciting opsonic and bactericidal antibodies (Gotto et al., 1993; Hellwig et al., 2003). This is also consistent with the increase of Prn-deficient strains in the aP-vaccinated era (Martin et al., 2015; Barkoff et al., 2019). Indeed, a Prn-negative strain colonized the respiratory tract of aP-immunized mice more efficiently than Prn-positive strain (Hegerle et al., 2014; Safarchi et al., 2015), likely because Prn-negative strains are not targeted by the bactericidal antibodies generated after aP vaccination and directed against Prn. After the introduction of aP vaccine containing Prn in Japan, an increase in the frequency of Prn-deficient strains was reported (up to 40%). However, the number of Prn-negative strains decreased significantly to 8% after change to the use of aP vaccines without Prn (Hiramatsu et al., 2017) suggesting that the loss of Prn could be a transient adaptation of the bacteria to the selective pressure of aP vaccines containing Prn. In a growth inhibition assay, Thiriard et al. (2020) show reduced inhibition of bacterial growth in the presence of NIBSC 06/140 serum with three recent Prn-negative strains compared to B1917 and it may be that anti-Prn antibodies are, at least in part, responsible for the inhibition of B1917 growth observed. Our results suggest that Prn is the only antigen to generate complement-mediated bactericidal antibodies after aP vaccination and that other antigens generate bactericidal antibodies after disease. We hypothesize that this would also be the case following wP vaccination.

No evidence was found to highlight a role for PTX in complement resistance or potential complement-mediated bactericidal activity of anti-PTX antibodies generated after aP-vaccination or natural infection. PTX has been shown to inhibit neutrophil recruitment and to be required for efficient early colonization in the lungs of mice (Kirimanjeswara et al., 2005). In Denmark, a PTX one-component vaccine has been used to replace wP vaccines, which shows efficacy against the disease and is still in use (Thierry-Carstensen et al., 2013). Antibodies directed against PTX neutralize the toxin (Acquaye-Seedah et al., 2018) and are important in providing protection against disease. Despite high *B. pertussis* colonization following *B. pertussis* challenge, infant baboons born to mothers vaccinated with the PTX mono-component vaccine show no disease symptoms, compared to baboons born to unvaccinated mothers that have the disease (Kapil et al., 2018). Another study reports that anti-PTX antibody hu1B7 is sufficient to prevent and treat clinical pertussis symptoms (Nguyen et al., 2020). These results suggest that PTX, which is mainly secreted, induces toxin-neutralizing antibodies which are sufficient to protect against pertussis disease.

The two adhesins, FHA and Fim, do not seem to be involved in complement resistance or elicit complement-mediated bactericidal antibodies after aP-vaccination or disease. These antigens are involved in adherence to the respiratory ciliated epithelial cells (Van Den Berg et al., 1999; Rodríguez et al., 2006). It was shown that anti-Fim and anti-FHA antibodies reduce attachment of bacteria to respiratory epithelial cells (Rodríguez et al., 2006) suggesting a bacterial adherence inhibitory role of these antibodies. Levels of IgG antibodies to FHA were found to be partially predictive for opsonophagocytosis of B1917 bacteria (Hovingh et al., 2018). FHA was reported to bind C4BP, an inhibitor of the classical pathway of complement activation (Berggård et al., 2001). Antibodies directed against FHA may thus prevent FHA binding to C4BP and so block protection to complement attack. It was reported that C4BP is also involved in the regulation of the alternative pathway by preventing non-desired C3 convertase formation (Blom, 2002), which could be a reason for the slightly increased sensitivity to complement alone observed with the KO FHA variant.

Our study confirms that Vag8 and BrkA are involved in complement resistance, but no evidence was found to show potential complement-mediated bactericidal activity of the anti-BrkA and anti-Vag8 antibodies generated after disease. Vaccination with recombinant Vag8 leads to the production of protective antibodies in a mouse model (De Gouw et al., 2014). The binding of Vag8 to the C1 esterase inhibitor prevents complement deposition on the bacterial surface by allowing the consumption of precursors of the C3 convertase, C4 and C2 (Hovingh et al., 2017). It was previously reported that anti-BrkA antibodies can boost the existing bactericidal capacity of human serum against *B. pertussis* by inhibiting BrkA (Oliver and Fernandez, 2001). BrkA is involved in complement resistance by preventing accumulation of deposited C4 and formation of the membrane attack complex (Barnes and Weiss, 2001). Vaccination of mice with recombinant BrkA alone failed to protect animals against *B. pertussis* colonization, but in contrast, when BrkA was included in a multicomponent vaccine containing PT and FHA, protection against colonization of mice occurred similar to using a tri-component vaccine including PT, FHA and Prn (Marr et al., 2008). As BrkA and Vag8 play a crucial role in complement resistance, it is possible that the antibodies generated after disease against these antigens only act to block complement protein binding. Indeed, it was observed that the passenger domain of Vag8, in addition of being inserted on the bacterial membrane and on outer membrane vesicles, is also secreted (Hovingh et al., 2017).

The toxin CyaA promotes *B. pertussis* colonization by inhibiting the clearance of the bacteria by phagocytic cells. It was shown that antibodies against this antigen induce phagocytosis by neutrophils (Weingart et al., 2000b) and that the RTX domain of CyaA was sufficient to generate toxin neutralizing antibodies (Wang and Maynard, 2015). In our study, CyaA does not seem to be involved in complement resistance or in generating complement-mediated bactericidal antibodies after disease.

TcfA has a cell associated and a secreted form and is required for the colonization and persistence of *B. pertussis* in mouse trachea (Finn and Stevens, 1995). Several clinical isolates have been reported to be deficient in TcfA due to gene deletion or mutation. Interestingly, TcfA is not produced by *B. parapertussis* and *B. bronchiseptica* (Van Gent et al., 2007). In our study, no evidence was found to support a role of TcfA in complement resistance or in the generation of complement-mediated bactericidal antibodies.

Convalescent samples elicit complement-mediated bactericidal activity for every single knock-out mutant generated in this study suggesting that antibodies targeting other *B. pertussis* antigens are bactericidal. It has been reported that antibodies directed against lipooligosaccharide (LOS) are bactericidal and result in a reduction in bacteria colonization in the lungs and trachea of mice (Mountzouros et al., 1992; Weiss et al., 1999). Absorption of serum using bacterial strains highlighted that antibodies directed against LOS or bvg-regulated proteins could have bactericidal activity (Weiss et al., 1999). Recently, Hovingh et al. (2018) showed that anti-LOS IgG antibodies are enhanced during convalescence. Moreover, serum bactericidal antibodies are induced after vaccination of mice with oligosaccharide conjugates (Kubler-Kielb et al., 2011). Another study reports that vaccination of mice with micelles induces high titers of bactericidal antibodies directed against OmpP, the major outer-membrane protein of *B. pertussis* (Van Loo et al., 2002). Several surface proteins have been identified to be immunogenic (Tefon et al., 2011) and could be analyzed to determine the function of these antibodies. Other antigens including the autotransporter BapC (Noofeli et al., 2011; Riaz et al., 2015) and the polysaccharide Bps (Ganguly et al., 2014) have been reported to be involved in complement resistance. Bps is required for colonization of the nose and trachea of mice and seems to be involved in biofilm formation in the nasopharynx (Conover et al., 2010). Future studies could generate single or multiple antigen deletion mutants, including LOS mutants to determine which antigens are required to induce complement-mediated bactericidal antibodies and could be included in a new generation aP vaccine.

In summary, we have demonstrated that Prn is the sole antigen in aP vaccines that generates complement-mediated bactericidal antibodies, whereas disease induces bactericidal antibodies which do not consist of anti-Prn alone. This powerful effect of Prn-containing aP vaccines is likely to be the main driver of the loss of Prn seen primarily in aP-using countries and which contributes to the sub-optimal protection provided by aP vaccines.

## Data Availability Statement

All datasets presented in this study are included in the article/Supplementary Material.

## Ethics Statement

The MULTIBOOST study was approved by the MHRA and the NRES Committee London – Brent, REC reference 13/LO/0681. The Eudract registration was 2012-005273-31. The SKI and Immfact studies were approved by the accredited Review Board METC UMC Utrecht. All participants provided written informed consent. For participants under 16, written informed consent was provided by parents or guardians of participants.

## Author Contributions

EL designed and performed the experiments, interpreted the data, prepared the figures, and wrote the manuscript. BC, IF-M, RP, and FA performed certain experiments. MM provided the ELISA data. CE provided the multiplex immunoassay data and reviewed the manuscript. AG coordinated the study, interpreted the data, and critically revised the figures and the manuscript. BC and ST interpreted the data and reviewed the figures and the manuscript. All authors approved the final version of the manuscript.

## Conflict of Interest

The authors declare that the research was conducted in the absence of any commercial or financial relationships that could be construed as a potential conflict of interest.

## Supplementary Material

FIGURE S1 Antibodies binding onto B1917 (WT) and knock-out variants deficient in each of the acellular vaccine antigens determined by flow cytometry. A and B. Quantification of total IgG binding onto B1917 (WT) and KO variants using 9 pre-aP **(A)** or post-aP **(B)** samples. Only sera from individuals who had received a Fim-containing vaccine (Repevax, *n* = 5) were tested for KO Fim3. The fluorescence intensity was determined from technical duplicates and reported relative to those of the WT strain. Geometric means with 95% confidence interval are represented. Statistical analyses were performed, and significant *p* values relative to WT strain are indicated (^∗∗^*p* ≤ 0.01, ^∗^*p* ≤ 0.05). **(C)** Quantification of anti-Prn IgG binding onto the complemented Prn (Comp Prn) strain. The fluorescence intensity was determined from two separate experiments with technical duplicates and means and standard deviation errors are given. Statistical analyses were performed, and significant *p* values relative to WT strain are indicated (^****^*p* ≤ 0.0001, ^∗^*p* ≤ 0.05). **(D)** Quantification of total IgG binding onto complemented Prn (Comp Prn) strain using 9 pre-aP or post-aP vaccine samples. The fluorescence intensity was determined from technical duplicates and reported relative to those of the WT strain. Geometric means with 95% confidence interval are represented. Statistical analyses were performed, and significant *p* values relative to WT strain are indicated (^∗∗^*p* ≤ 0.01).

FIGURE S2 Determination of antibodies binding onto B1917 (WT) and knock-out variants using convalescent samples. **(A,B)** Quantification of total IgG binding onto B1917 (WT) and KO variants using the 1st WHO International Standard pertussis antiserum NIBSC 06/140 **(A)** and 10 convalescent **(B)** samples. The fluorescence intensity was determined from at least two experiments with technical duplicates for panel **(A)** and at least once for panel **(B)** and reported relative to those of the WT strain. Geometric means with 95% confidence interval are represented. Statistical analyses were performed, and significant *p* values relative to WT strain are indicated (^****^*p* ≤ 0.0001, ^∗∗∗^*p* ≤ 0.001).

FIGURE S3 Serum bactericidal activity of post-aP vaccine samples with B1917 (WT) and KO variants deficient of vaccine candidate antigens. Serum bactericidal titers were assigned as the interpolated serum dilution which gives 50% of bacterial killing when incubating the bacteria with serial dilutions of post-aP vaccine serum and active complement. 7 sera were analyzed. The geometric means with 95% confidence interval are represented. Statistical analyses were performed for each data point using the corresponding WT data point as a control, and no significant *p* values were determined.

TABLE S1 Sequences of the primers used to generate knock-out mutants and pertactin complemented strain in B1917.

## References

[B1] Acquaye-Seedah E. Reczek E. E. Russell H. H. DiVenere A. M. Sandman S. O. Collins J. H. (2018). Characterization of individual human antibodies that bind pertussis toxin stimulated by acellular immunization. *Infect. Immun.* 86 1–17. 10.1128/IAI.00004-18 29581192 PMC5964521

[B2] Althouse B. M. Scarpino S. V. (2015). Asymptomatic transmission and the resurgence of *Bordetella pertussis*. *BMC Med.* 13:146. 10.1186/s12916-015-0382-8 26103968 PMC4482312

[B3] Amdahl H. Jarva H. Haanperä M. Mertsola J. He Q. Jokiranta T. S. (2011). Interactions between *Bordetella pertussis* and the complement inhibitor factor H. *Mol. Immunol.* 48 697–705. 10.1016/j.molimm.2010.11.015 21167605

[B4] Barkoff A. M. Gröndahl-Yli-Hannuksela K. He Q. (2015). Seroprevalence studies of pertussis: what have we learned from different immunized populations. *Pathog. Dis.* 73 1–12. 10.1093/femspd/ftv050 26208655

[B5] Barkoff A. M. Mertsola J. Pierard D. Dalby T. Hoegh S. V. Guillot S. (2019). Pertactin-deficient *Bordetella pertussis* isolates: evidence of increased circulation in Europe, 1998 to 2015. *Eurosurveillance* 24 1–11. 10.2807/1560-7917.ES.2019.24.7.1700832 30782265 PMC6381657

[B6] Barnes M. G. Weiss A. A. (2001). BrkA protein of *Bordetella pertussis* inhibits the classical pathway of complement after C1 deposition. *Infect. Immun.* 69 3067–3072. 10.1128/IAI.69.5.3067 11292725 PMC98261

[B7] Barnes M. G. Weiss A. A. (2002). Growth phase influences complement resistance of *Bordetella pertussis*. *Infect. Immun.* 70 403–406. 10.1128/IAI.70.1.403-406.2002 11748208 PMC127634

[B8] Bart M. J. Harris S. R. Advani A. Arakawa Y. Bottero D. Bouchez V. (2014a). Global population structure and evolution of *Bordetella pertussis* and their relationship with vaccination. *mBio* 5 1–13. 10.1128/mBio.01074-14 24757216 PMC3994516

[B9] Bart M. J. Zeddeman A. van der Heide H. G. J. Heuvelman K. van Gent M. Mooi R. (2014b). Complete genome sequences of *Bordetella pertussis* isolates B1917 and B1920, representing two predominant global lineages. *Genome Announc.* 2 e1301–e1314. 10.1128/genomeA.01301-14. 25540342 PMC4276820

[B10] Belcher T. Preston A. (2015). *Bordetella pertussis* evolution in the (functional) genomics era. *Pathog. Dis.* 73:ftv064. 10.1093/femspd/ftv064 26297914 PMC4626590

[B11] Berggård K. Lindahl G. Dahlbäck B. Blom A. M. (2001). *Bordetella pertussis* binds to human C4b-binding protein (C4BP) at a site similar to that used by the natural ligand C4b. *Eur. J. Immunol.* 31 2771–2780. 10.1002/1521-4141(200109)31:9<2771::aid-immu2771>3.0.co;2-0 11536176

[B12] Blom A. M. (2002). Structural and functional studies of complement inhibitor C4b-binding protein. *Biochem. Soc. Trans.* 30 978–982. 10.1042/BST0300978 12440957

[B13] Bouchez V. Hegerle N. Strati F. Njamkepo E. Guiso N. (2015). New data on vaccine antigen deficient *Bordetella pertussis* isolates. *Vaccines* 3 751–770. 10.3390/vaccines3030751 26389958 PMC4586476

[B14] Brookes C. Freire-Martin I. Cavell B. Alexander F. Taylor S. Persaud R. (2018). *Bordetella pertussis* isolates vary in their interactions with human complement components article. *Emerg. Microbes Infect.* 7:81. 10.1038/s41426-018-0084-3 29739922 PMC5940884

[B15] Brookes C. Kuisma E. Alexander F. Allen L. Tipton T. Ram S. (2013). Development of a large scale human complement source for use in bacterial immunoassays. *J. Immunol. Methods* 391 39–49. 10.1016/j.jim.2013.02.007 23485926

[B16] Carbonetti N. H. Artamonova G. V. Andreasen C. Bushar N. (2005). Pertussis toxin and adenylate cyclase toxin provide a one-two punch for establishment of. *Infect. Immun.* 73 2698–2703. 10.1128/IAI.73.5.2698 15845471 PMC1087369

[B17] Carbonetti N. H. Artamonova G. V. Andreasen C. Dudley E. Mays R. M. Worthington Z. E. V. (2004). Suppression of serum antibody responses by pertussis toxin after respiratory tract colonization by *Bordetella pertussis* and identification of an immunodominant lipoprotein. *Infect. Immun.* 72 3350–3358. 10.1128/IAI.72.6.3350-3358.2004 15155640 PMC415701

[B18] Conover M. S. Sloan G. P. Love C. F. Sukumar N. Deora R. (2010). The Bps polysaccharide of *Bordetella pertussis* promotes colonization and biofilm formation in the nose by functioning as an adhesin. *Mol. Microbiol.* 23 1–7. 10.1161/CIRCULATIONAHA.110.956839 20633227 PMC2939936

[B19] da Silva Antunes R. Babor M. Carpenter C. Khalil N. Cortese M. Mentzer A. J. (2018). Th1/Th17 polarization persists following whole-cell pertussis vaccination despite repeated acellular boosters. *J. Clin. Invest.* 128 3853–3865. 10.1172/JCI121309 29920186 PMC6118631

[B20] De Gouw D. De Jonge M. I. Hermans P. W. M. Wessels H. J. C. T. Zomer A. Berends A. (2014). Proteomics-identified Bvg-activated autotransporters protect against *Bordetella pertussis* in a mouse model. *PLoS One* 9:e105011. 10.1371/journal.pone.0105011 25133400 PMC4136822

[B21] de Gouw D. Diavatopoulos D. A. Bootsma H. J. Hermans P. W. M. Mooi F. R. (2011). Pertussis: a matter of immune modulation. *FEMS Microbiol. Rev.* 35 441–474. 10.1111/j.1574-6976.2010.00257.x 21204863

[B22] Diavatopoulos D. A. Mills K. H. G. Kester K. E. Kampmann B. Silerova M. Heininger U. (2019). PERISCOPE: road towards effective control of pertussis. *Lancet Infect. Dis.* 19 e179–e186. 10.1016/S1473-3099(18)30646-7 30503084

[B23] Esposito S. Stefanelli P. Fry N. K. Fedele G. He Q. Paterson P. (2019). Pertussis prevention : reasons for resurgence, and differences in the current acellular pertussis vaccines. *Front. Immunol.* 10:1344. 10.3389/fimmu.2019.01344 31333640 PMC6616129

[B24] Fernandez R. C. Weiss A. A. (1994). Cloning and sequencing of a *Bordetella pertussis* serum resistance locus. *Infect. Immun.* 62 4727–4738. 10.1128/iai.62.11.4727-4738.1994 7927748 PMC303180

[B25] Findlow J. Balmer P. Borrow R. (2019). A review of complement sources used in serum bactericidal assays for evaluating immune responses to meningococcal ACWY conjugate vaccines. *Hum. Vaccines Immunother.* 15 2491–2500. 10.1080/21645515.2019.1593082 30883271 PMC6816443

[B26] Finn T. M. Stevens L. A. (1995). Tracheal colonization factor: a *Bordetella pertussis* secreted virulence determinant. *Mol. Microbiol.* 16 625–634. 10.1111/j.1365-2958.1995.tb02425.x 7476158

[B27] Fothergill L. D. Wright J. (1933). Influenzal meningitis the relation of age incidence to the bactericidal power of blood against the causal organism. *J. Immunol.* 24 273–284.

[B28] Ganguly T. Johnson J. B. Kock N. D. Parks G. D. Deora R. (2014). The *Bordetella pertussis* Bps polysaccharide enhances lung colonization by conferring protection from complement-mediated killing. *Cell. Microbiol.* 16 1105–1118. 10.1111/cmi.12264 24438122 PMC4065191

[B29] Geurtsen J. Fae K. C. Van Den Dobbelsteen G. P. J. M. (2014). Importance of (antibody-dependent) complement-mediated serum killing in protection against *Bordetella pertussis*. *Expert Rev. Vaccines* 13 1229–1240. 10.1586/14760584.2014.944901 25081731

[B30] Gill C. Ram S. Welsch J. DeTora L. Anemona A. (2011). Correlation between serum bactericidal activity against *Neisseria meningitidis* serogroups A, C, W-135 and Y measured using human versus rabbit serum as the complement source. *Vaccine* 30 29–34. 10.1038/jid.2014.371 22075087 PMC3906633

[B31] Goldschneider I. Gotschlich E. C. Artenstein M. S. (1969). Human immunity to the meningococcus. I. The role of humoral antibodies. *J. Exp. Med.* 129 1307–1326. 10.1084/jem.129.6.1307 4977280 PMC2138650

[B32] Gotto J. W. Eckhardt T. Reilly P. A. Scott J. V. Cowell J. L. Metcalf T. N. (1993). Biochemical and immunological properties of two forms of pertactin, the 69,000-molecular-weight outer membrane protein of *Bordetella pertussis*. *Infect. Immun.* 61 2211–2215. 10.1128/iai.61.5.2211-2215.1993 8478113 PMC280825

[B33] Gustafsson L. Hallander H. O. Olin P. Reizenstein E. Storsaeter J. (1996). A controlled trial of a two-component acellular, a five-component acellular, and a whole-cell pertussis vaccine. *N. Engl. J. Med.* 334 349–355. 10.1056/NEJM199602083340602 8538705

[B34] Hegerle N. Dore G. Guiso N. (2014). Pertactin deficient *Bordetella pertussis* present a better fitness in mice immunized with an acellular pertussis vaccine. *Vaccine* 32 6597–6600. 10.1016/j.vaccine.2014.09.068 25312274

[B35] Hellwig S. M. M. Rodriguez M. E. Berbers G. A. M. van de Winkel J. G. J. Mooi F. R. (2003). Crucial role of antibodies to pertactin in *Bordetella pertussis* immunity. *J. Infect. Dis.* 188 738–742. 10.1086/377283 12934190

[B36] Higgs R. Higgins S. C. Ross P. J. Mills K. H. G. (2012). Immunity to the respiratory pathogen *Bordetella pertussis*. *Mucosal Immunol.* 5 485–500. 10.1038/mi.2012.54 22718262

[B37] Hiramatsu Y. Miyaji Y. Otsuka N. Arakawa Y. Shibayama K. Kamachi K. (2017). Significant decrease in pertactin-deficient *Bordetella pertussis* isolates. *Jpn. Emerg. Infect. Dis.* 23 699–701. 10.3201/eid2304.161575 28322702 PMC5367402

[B38] Hovingh E. S. Kuipers B. Bonaèiæ Marinoviæ A. A. Jan Hamstra H. Hijdra D. Mughini Gras L. (2018). Detection of opsonizing antibodies directed against a recently circulating *Bordetella pertussis* strain in paired plasma samples from symptomatic and recovered pertussis patients. *Sci. Rep.* 8 1–11. 10.1038/s41598-018-30558-8 30104573 PMC6089961

[B39] Hovingh E. S. van den Broek B. Kuipers B. Pinelli E. Rooijakkers S. H. M. Jongerius I. (2017). Acquisition of C1 inhibitor by *Bordetella pertussis* virulence associated gene 8 results in C2 and C4 consumption away from the bacterial surface. *PLoS Pathog.* 13:e1006531. 10.1371/journal.ppat.1006531 28742139 PMC5542704

[B40] Jongerius I. Schuijt T. J. Mooi F. R. Pinelli E. (2015). Complement evasion by *Bordetella pertussis*: implications for improving current vaccines. *J. Mol. Med.* 93 395–402. 10.1007/s00109-015-1259-1 25686752 PMC4366546

[B41] Kapil P. Papin J. F. Wolf R. F. Zimmerman L. I. Wagner L. D. Merkel T. J. (2018). Maternal vaccination with a monocomponent pertussis toxoid vaccine is sufficient to protect infants in a baboon model of whooping cough. *J. Infect. Dis.* 217 1231–1236. 10.1093/infdis/jiy022 29346585 PMC6018939

[B42] Kirimanjeswara G. S. Agosto L. M. Kennett M. J. Bjornstad O. N. Harvill E. T. (2005). Pertussis toxin inhibits neutrophil recruitment to delay antibody-mediated clearance of *Bordetella pertussis*. *J. Clin. Invest.* 115 3594–3601. 10.1172/JCI24609.3594 16294220 PMC1283938

[B43] Klein N. P. Bartlett J. Fireman B. Baxter R. (2016). Waning Tdap effectiveness in adolescents. *Pediatrics* 137:e20153326. 10.1542/peds.2015-3326 26908667

[B44] Kubler-Kielb J. Vinogradov E. Lagergård T. Ginzberg A. King J. D. Preston A. (2011). Oligosaccharide conjugates of *Bordetella pertussis* and bronchiseptica induce bactericidal antibodies, an addition to pertussis vaccine. *Proc. Natl. Acad. Sci. U.S.A.* 108 4087–4092. 10.1073/pnas.1100782108 21367691 PMC3054038

[B45] Ladhani S. N. Andrews N. J. Southern J. Jones C. E. Amirthalingam G. Waight P. A. (2015). Antibody responses after primary immunization in infants born to women receiving a Pertussis-containing vaccine during pregnancy: single arm observational study with a historical comparator. *Clin. Infect. Dis.* 61 1637–1644. 10.1093/cid/civ695 26374816

[B46] Lam C. Octavia S. Ricafort L. Sintchenko V. Gilbert G. L. Wood N. (2014). Rapid increase in pertactin-deficient *Bordetella pertussis* isolates. *Australia. Emerg. Infect. Dis.* 20 626–633. 10.3201/eid2004.131478 24655754 PMC3966384

[B47] Lamm M. E. (1997). Interaction of antigens and antibodies at mucosal surfaces. *Annu. Rev. Microbiol.* 51 311–340. 10.1146/annurev.micro.51.1.311 9343353

[B48] Locht C. Antoine R. Jacob-Dubuisson F. (2001). *Bordetella pertussis*, molecular pathogenesis under multiple aspects. *Curr. Opin. Microbiol.* 4 82–89. 10.1016/S1369-5274(00)00169-7 11173039

[B49] Luu L. D. W. Octavia S. Aitken C. Zhong L. Raftery M. J. Sintchenko V. (2020). Surfaceome analysis of Australian epidemic *Bordetella pertussis* reveals potential vaccine antigens. *Vaccine* 38 539–548. 10.1016/j.vaccine.2019.10.062 31703933

[B50] Marr N. Oliver D. C. Laurent V. Poolman J. Denoël P. Fernandez R. C. (2008). Protective activity of the *Bordetella pertussis* BrkA autotransporter in the murine lung colonization model. *Vaccine* 26 4306–4311. 10.1016/j.vaccine.2008.06.017 18582518

[B51] Marr N. Shah N. R. Lee R. Kim E. J. Fernandez R. C. (2011). *Bordetella pertussis* autotransporter Vag8 binds human C1 esterase inhibitor and confers serum resistance. *PLoS One* 6:e20585. 10.1371/journal.pone.0020585 21695123 PMC3114845

[B52] Martin S. W. Pawloski L. Williams M. Weening K. Debolt C. Qin X. (2015). Pertactin-negative *bordetella pertussis* strains: evidence for a possible selective advantage. *Clin. Infect. Dis.* 60 223–227. 10.1093/cid/ciu788 25301209

[B53] Mooi F. R. Jansen W. H. Brunings H. Gielen H. van der Heide H. G. J. Walvoort H. C. (1992). Construction and analysis of *Bordetella pertussis* mutants defective in the production of fimbriae. *Microb. Pathog.* 12 127–135. 10.1016/0882-4010(92)90115-5 1350044

[B54] Mountzouros K. T. Kimura A. Cowell J. L. (1992). A bactericidal monoclonal antibody specific for the lipooligosaccharide of *Bordetella pertussis* reduces colonization of the respiratory tract of mice after aerosol infection with *B. pertussis*. *Infect. Immun.* 60 5316–5318. 10.1128/iai.60.12.5316-5318.1992 1452367 PMC258314

[B55] Nguyen A. W. DiVenere A. M. Papin J. F. Connelly S. Kaleko M. Maynard J. A. (2020). Neutralization of pertussis toxin by a single antibody prevents clinical pertussis in neonatal baboons. *Sci. Adv.* 6 1–10. 10.1126/sciadv.aay9258 32076653 PMC7002138

[B56] Nieves D. J. Heininger U. (2016). *Bordetella pertussis*. *Mol. Med. Microbiol. Second Ed.* 3 1507–1527. 10.1016/B978-0-12-397169-2.00085-8 27337481

[B57] Noofeli M. Bokhari H. Blackburn P. Roberts M. Coote J. G. Parton R. (2011). BapC autotransporter protein is a virulence determinant of *Bordetella pertussis*. *Microb. Pathog.* 51 169–177. 10.1016/j.micpath.2011.04.004 21554944

[B58] Olin P. Hallander H. O. Gustafsson L. Reizenstein E. Storsaeter J. (2001). How to make sense of pertussis immunogenicity data. *Clin. Infect. Dis.* 33 S288–S291. 10.1086/322564 11709761

[B59] Oliver D. C. Fernandez R. C. (2001). Antibodies to BrkA augment killing of *Bordetella pertussis*. *Vaccine* 20 235–241. 10.1016/S0264-410X(01)00269-9 11567769

[B60] Persson C. Erjefält I. Alkner U. Baumgarten C. Greiff L. Gustafsson B. (1991). Plasma exudation as a first line respiratory mucosal defence. *Clin. Exp. Allergy* 21 17–24. 10.1111/j.1365-2222.1991.tb00799.x 2021873

[B61] Pinto M. V. Merkel T. J. (2017). Pertussis disease and transmission and host responses: insights from the baboon model of pertussis. *J. Infect.* 74 S114–S119. 10.1016/S0163-4453(17)30201-3 28646950

[B62] Plotkin S. A. (2020). Updates on immunologic correlates of vaccine-induced protection. *Vaccine* 38 2250–2257. 10.1016/j.vaccine.2019.10.046 31767462

[B63] Riaz M. R. Siddiqi A. R. Bokhari H. (2015). Structural and functional studies of BapC protein of *Bordetella pertussis*. *Microbiol. Res.* 174 56–61. 10.1016/j.micres.2015.03.006 25946329

[B64] Roberts M. Tite J. P. Fairweather N. F. Dougan G. Charles I. G. (1992). Recombinant P.69/pertactin: immunogenicity and protection of mice against *Bordetella pertussis* infection. *Vaccine* 10 43–48. 10.1016/0264-410X(92)90418-J 1539459

[B65] Rodríguez M. E. Hellwig S. M. M. Pérez Vidakovics M. L. A. Berbers G. A. M. Van De Winkel J. G. J. (2006). *Bordetella pertussis* attachment to respiratory epithelial cells can be impaired by fimbriae-specific antibodies. *FEMS Immunol. Med. Microbiol.* 46 39–47. 10.1111/j.1574-695X.2005.00001.x 16420595

[B66] Ross P. J. Sutton C. E. Higgins S. Allen A. C. Walsh K. Misiak A. (2013). Relative contribution of Th1 and Th17 cells in adaptive immunity to *Bordetella pertussis*: towards the rational design of an improved acellular pertussis vaccine. *PLoS Pathog.* 9:e1003264. 10.1371/journal.ppat.1003264 23592988 PMC3617212

[B67] Safarchi A. Octavia S. Luu L. D. W. Tay C. Y. Sintchenko V. Wood N. (2015). Pertactin negative *Bordetella pertussis* demonstrates higher fitness under vaccine selection pressure in a mixed infection model. *Vaccine* 33 6277–6281. 10.1016/j.vaccine.2015.09.064 26432908

[B68] Santos G. F. Deck R. R. Donnelly J. Blackwelder W. Granoff D. M. (2001). Importance of complement source in measuring meningococcal bactericidal titers. *Clin. Diagn. Lab. Immunol.* 8 616–623. 10.1128/CDLI.8.3.616-623.2001 11329468 PMC96111

[B69] Sebo P. Osicka R. Masin J. (2014). Adenylate cyclase toxin-hemolysin relevance for pertussis vaccines. *Expert Rev. Vaccines* 13 1215–1227. 10.1586/14760584.2014.944900 25090574

[B70] Tefon B. E. Maaß S. Özcengiz E. Becher D. Hecker M. Özcengiz G. (2011). A comprehensive analysis of *Bordetella pertussis* surface proteome and identification of new immunogenic proteins. *Vaccine* 29 3583–3595. 10.1016/j.vaccine.2011.02.086 21397717

[B71] Thalen M. Van Den Ijssel J. Jiskoot W. Zomer B. Roholl P. De Gooijer C. (1999). Rational medium design for *Bordetella pertussis*: basic metabolism. *J. Biotechnol.* 75 147–159. 10.1016/S0168-1656(99)00155-8 10553654

[B72] Thierry-Carstensen B. Dalby T. Stevner M. A. Robbins J. B. Schneerson R. Trollfors B. (2013). Experience with monocomponent acellular pertussis combination vaccines for infants, children, adolescents and adults-A review of safety, immunogenicity, efficacy and effectiveness studies and 15 years of field experience. *Vaccine* 31 5178–5191. 10.1016/j.vaccine.2013.08.034 23994021

[B73] Thiriard A. Raze D. Locht C. (2020). Development and standardization of a high-throughput *Bordetella pertussis* growth-inhibition assay. *Front. Microbiol.* 11:777. 10.3389/fmicb.2020.00777 32425912 PMC7212404

[B74] Van De Waterbeemd B. Streefland M. Pennings J. Van Der Pol L. Beuvery C. Tramper J. (2009). Gene-expression-based quality scores indicate optimal harvest point in *Bordetella pertussis* cultivation for vaccine production. *Biotechnol. Bioeng.* 103 900–908. 10.1002/bit.22326 19405154

[B75] Van Den Berg B. M. Beekhuizen H. Willems R. J. L. Mooi F. R. Van Furth R. (1999). Role of *Bordetella pertussis* virulence factors in adherence to epithelial cell lines derived from the human respiratory tract. *Infect. Immun.* 67 1056–1062. 10.1128/iai.67.3.1056-1062.1999 10024543 PMC96429

[B76] van der Lee S. Hendrikx L. H. Sanders E. A. M. Berbers G. A. M. Buisman A. M. (2018). Whole-cell or acellular pertussis primary immunizations in infancy determines adolescent cellular immune profiles. *Front. Immunol.* 9:51. 10.3389/fimmu.2018.00051 29416544 PMC5787539

[B77] Van Gent M. Pierard D. Lauwers S. Van Der Heide H. G. J. King A. J. Mooi F. R. (2007). Characterization of *Bordetella pertussis* clinical isolates that do not express the tracheal colonization factor. *FEMS Immunol. Med. Microbiol.* 51 149–154. 10.1111/j.1574-695X.2007.00291.x 17854476

[B78] Van Loo I. H. M. Heuvelman K. J. King A. J. Mooi F. R. (2002). Multilocus sequence typing of *Bordetella pertussis* based on surface protein genes. *J. Clin. Microbiol.* 40 1994–2001. 10.1128/JCM.40.6.1994-2001.2002 12037054 PMC130760

[B79] Van Twillert I. Marinoviæ A. A. B. Kuipers B. van Gaans-van Den Brink J. A. M. Sanders E. A. M. Van Els C. A. C. M. (2017). Impact of age and vaccination history on long-Term serological responses after symptomatic B. Pertussis infection, a high dimensional data analysis. *Sci. Rep.* 7:40328. 10.1038/srep40328 28091579 PMC5238437

[B80] Wang X. Maynard J. A. (2015). The Bordetella adenylate cyclase repeat-in-toxin (RTX) domain is immunodominant and elicits neutralizing antibodies. *J. Biol. Chem.* 290 3576–3591. 10.1074/jbc.M114.585281 25505186 PMC4319024

[B81] Warfel J. M. Zimmerman L. I. Merkel T. J. (2014). Acellular pertussis vaccines protect against disease butfail to prevent infection and transmission ina nonhuman primate model. *Proc. Natl. Acad. Sci. U.S.A.* 111 787–792. 10.1073/pnas.1314688110 24277828 PMC3896208

[B82] Weigand M. R. Pawloski L. C. Peng Y. Ju H. Burroughs M. Cassiday P. K. (2018). Screening and genomic characterization of filamentous hemagglutinin-deficient *Bordetella pertussis*. *Infect. Immun.* 86 5–7. 10.1128/IAI.00869-17 29358336 PMC5865017

[B83] Weingart C. L. Keitel W. A. Edwards K. M. Weiss A. A. (2000a). Characterization of bactericidal immune responses following vaccination with acellular pertussis vaccines in adults. *Infect. Immun.* 68 7175–7179. 10.1128/IAI.68.12.7175-7179.2000 11083851 PMC97836

[B84] Weingart C. L. Mobberley-Schuman P. S. Hewlett E. L. Gray M. C. Weiss A. A. (2000b). Neutralizing antibodies to adenylate cyclase toxin promote phagocytosis of *Bordetella pertussis* by human neutrophils. *Infect. Immun.* 68 7152–7155. 10.1128/IAI.68.12.7152-7155.2000 11083845 PMC97830

[B85] Weiss A. A. Mobberley P. S. Fernandez R. C. Mink C. M. (1999). Characterization of human bactericidal antibodies to *Bordetella pertussis*. *Infect. Immun.* 67 1424–1431. 10.1128/iai.67.3.1424-1431.1999 10024590 PMC96476

[B86] Wilk M. M. Borkner L. Misiak A. Curham L. Allen A. C. Mills K. H. G. (2019). Immunization with whole cell but not acellular pertussis vaccines primes CD4 TRM cells that sustain protective immunity against nasal colonization with *Bordetella pertussis*. *Emerg. Microbes Infect.* 8 169–185. 10.1080/22221751.2018.1564630 30866771 PMC6455184

[B87] Williams M. M. Sen K. A. Weigand M. R. Skoff T. H. Cunningham V. A. Halse T. A. (2016). *Bordetella pertussis* strain lacking pertactin and pertussis toxin. *Emerg. Infect. Dis.* 22 319–322. 10.3201/eid2202.151332 26812174 PMC4734536

[B88] Witt M. A. Arias L. Katz P. H. Truong E. T. Witt D. J. (2013). Reduced risk of pertussis among persons ever vaccinated with whole cell pertussis vaccine compared to recipients of acellular pertussis vaccines in a large US cohort. *Clin. Infect. Dis.* 56 1248–1254. 10.1093/cid/cit046 23487373

[B89] Xu Y. Liu B. Gröndahl-Yli-Hannuksila K. Tan Y. Feng L. Kallonen T. (2015). Whole-genome sequencing reveals the effect of vaccination on the evolution of *Bordetella pertussis*. *Sci. Rep.* 5 1–10. 10.1038/srep12888 26283022 PMC4539551

[B90] Yeung K. H. T. Duclos P. Nelson E. A. S. Hutubessy R. C. W. (2017). An update of the global burden of pertussis in children younger than 5 years: a modeling study. *Lancet Infect. Dis.* 17 974–980. 10.1016/S1473-3099(17)30390-0 28623146

