# Supplementary material for: Acellular Pertussis Vaccines Induce Anti-pertactin Bactericidal Antibodies Which Drives the Emergence of Pertactin-Negative Strains
Source: Front Microbiol. 2020 Aug 27;11:2108. doi: 10.3389/fmicb.2020.02108 (PMC7481377; doi:10.3389/fmicb.2020.02108)
Supplement: FIGURE S1 — Antibodies binding onto B1917 (WT) and knock-out variants deficient in each of the acellular vaccine antigens determined by flow cytometry. A and B. Quantification of total IgG binding onto B1917 (WT) and KO variants using 9 pre-aP (A) or post-aP (B) samples. Only sera from individuals who had received a Fim-containing vaccine (Repevax, n = 5) were tested for KO Fim3. The fluorescence intensity was determined from technical duplicates and reported relative to those of the WT strain. Geometric means with 95% confidence interval are represented. Statistical analyses were performed, and significant p values relative to WT strain are indicated (∗∗p ≤ 0.01, ∗p ≤ 0.05). (C) Quantification of anti-Prn IgG binding onto the complemented Prn (Comp Prn) strain. The fluorescence intensity was determined from two separate experiments with technical duplicates and means and standard deviation errors are given. Statistical analyses were performed, and significant p values relative to WT strain are indicated (****p ≤ 0.0001, ∗p ≤ 0.05). (D) Quantification of total IgG binding onto complemented Prn (Comp Prn) strain using 9 pre-aP or post-aP vaccine samples. The fluorescence intensity was determined from technical duplicates and reported relative to those of the WT strain. Geometric means with 95% confidence interval are represented. Statistical analyses were performed, and significant p values relative to WT strain are indicated (∗∗p ≤ 0.01). [file Data_Sheet_1.DOCX]

Supplementary Material

| **Primers** | **Sequences** |
| --- | --- |
| fim3-L Fw | aaaaggtctctCGAGCATAACGCTCCCAACCCTGT |
| fim3-L Rv | aaaaggtctccATGTGGTGATTCTGATACTGAGGGGT |
| fim3-R Fw | aaaaggtctccACATTTTTCCGTCGTCTACCCCTG |
| fim3-R Rv | aaaaggtctcgAACTACCAGATGCTGGAAAAGCGT |
| prn-L Fw | aaaaggtctctCGAGGGCGGCGAAGAACTGGAATA |
| prn-L Rv | aaaaggtctccATGTAGCGAGGAGGGTCTATCCCC |
| prn-R Fw | aaaaggtctccACATTGGATGCCAGGTGGAGAGCA |
| prn-R Rv | aaaaggtctcgAACTATCCGGCAGCAGTCTGAACA |
| fha-L Fw | aaaaggtctctCGAGCGATGTCGAGTACGACGAGG |
| fha-L Rv | aaaaggtctctATGTATTCCGACCAGCGAAGTGAA |
| fha-R Fw | aaaaggtctccACATATAGGTAGTCGCGGCCTGC |
| fha-R Rv | aaaaggtctcgAACTTCATGATTCAAGAATCGCGGC |
| ptx-L Fw | aaaaggtctctCGAGAGGGAACCGACCCCAAGATA |
| ptx-L Rv | aaaaggtctccATGTGAATTGCCCGAGTGCAACG |
| ptx-R Fw | aaaaggtctccACATAGCGTCGATATGTTGAGCCG |
| ptx-R Rv | aaaaggtctcgAACTCAATGCCACGGGAAACAAGG |
| vag8-L-Fw | aaaaggtctcgAACTAGGGCAGGCTGTACGAAGAC |
| vag8-L-Rv | aaaaggtctccACATCCAATGGCAATATCGTTGAA |
| vag8-R-Fw | aaaaggtctccATGTCATGCCTTCCTGCACATAGA |
| vag8-R-Rv | aaaaggtctctCGAGGCACGGTATCAACGTGACTG |
| brkA-L-Fw | aaaaggtctctCGAGTGAACACCAGCACGCATATC |
| brkA-L-Rv | aaaaggtctccATGTGTGCCACCAAAAGAGAAGTT |
| brkA-R-Fw | aaaaggtctccACATCCAATGAAAAACCCCGCCAG |
| brkA-R-Rv | aaaaggtctcgAACTAAGGAAAAGAAGGCTCGCGT |
| tcfA-L-Fw | aaaaggtctctCGAGCTACTTTCCCAGCCAGAGCG |
| tcfA -L-Rv | aaaaggtctccATGTGTGCCGCAGATGTCAGTCC |
| tcfA -R-Fw | aaaaggtctccACATAGGTTCTGGTCTCAAGGGGA |
| tcfA -R-Fw | aaaaggtctcgAACTCTGGTAGCGCATCGTATGGC |
| cyaC-Fw | CCGCAGTTCCAGGCATTC |
| cyaC-Rv | GCTGCATGTGGATCTGTCG |
| cyaB-Fw | CTGTCAACTGGCGCTGAAG |
| cyaB-Rv | ATCCAGCTTGCTGCTGGTATG |
| Over_cyaC-Fw | aaggaactcgagCCGCAGTTCCAGGCATTC |
| Over_cyaB-Rv | aaccaagaattcATCCAGCTTGCTGCTGGTATG |
| PCR8Gw_SpeI-UP | CGCCCTTAAAAGAACTAGTGACCCTGTGTATAAGGGAGCC |
| PCR8Gw_SpeI-LOW | GGCTCCCTTATACACAGGGTCACTAGTTCTTTTAAGGGCG |
| PCR8Gw_HindIII-UP | GCCTAATGCGGCCGCGAAGCTTGTACCGGATCCAGTCGACTG |
| PCR8Gw_HindIII-LOW | CAGTCGACTGGATCCGGTACAAGCTTCGCGGCCGCATTAGGC |
| Prn_comp_SpeI | aaACTAGTACTGCCAAGACGGTATCTGT |
| Prn_comp_BglII | aaAAGCTTGGGATAGACCCTCCTCGCTT |

**Supplementary Table 1.** Sequences of the primers used to generate knock-out mutants and pertactin complemented strain in B1917.

**
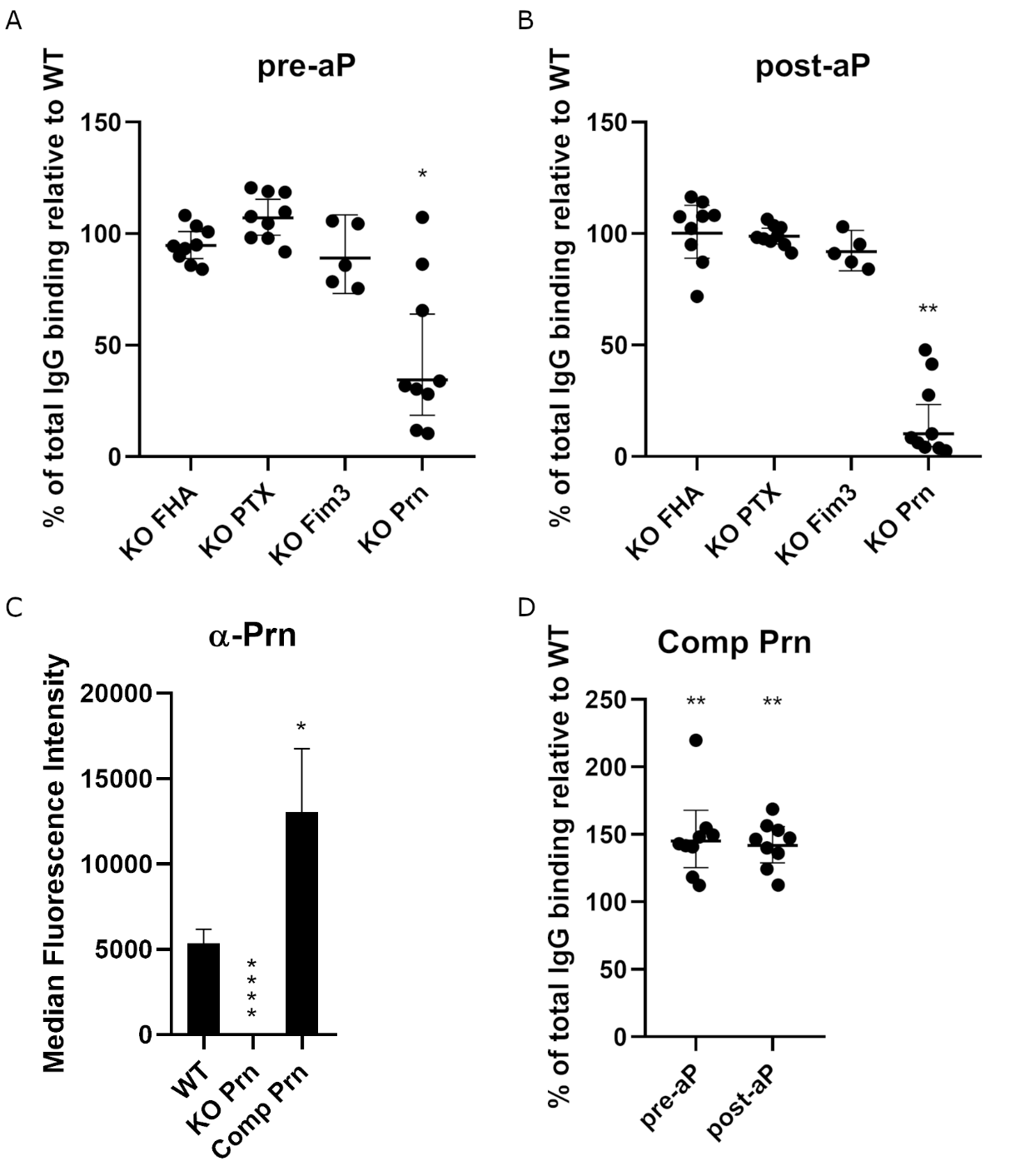
**

**Supplementary Figure 1.** **Antibodies binding onto B1917 (WT) and knock-out variants deficient in each of the acellular vaccine antigens determined by flow cytometry.** A and B. Quantification of total IgG binding onto B1917 (WT) and KO variants using 9 pre-aP (A) or post-aP (B) samples. Only sera from individuals who had received a Fim-containing vaccine (Repevax, n=5) were tested for KO Fim3. The fluorescence intensity was determined from technical duplicates and reported relative to those of the WT strain. Geometric means with 95% confidence interval are represented. Statistical analyses were performed, and significant p values relative to WT strain are indicated (** for p ≤ 0.01, * for p ≤ 0.05). C. Quantification of anti-Prn IgG binding onto the complemented Prn (Comp Prn) strain. The fluorescence intensity was determined from two separate experiments with technical duplicates and means and standard deviation errors are given. Statistical analyses were performed, and significant p values relative to WT strain are indicated (**** for p ≤ 0.0001, * for p ≤ 0.05). D. Quantification of total IgG binding onto complemented Prn (Comp Prn) strain using 9 pre-aP or post-aP vaccine samples. The fluorescence intensity was determined from technical duplicates and reported relative to those of the WT strain. Geometric means with 95% confidence interval are represented. Statistical analyses were performed, and significant p values relative to WT strain are indicated (** for p ≤ 0.01).

**
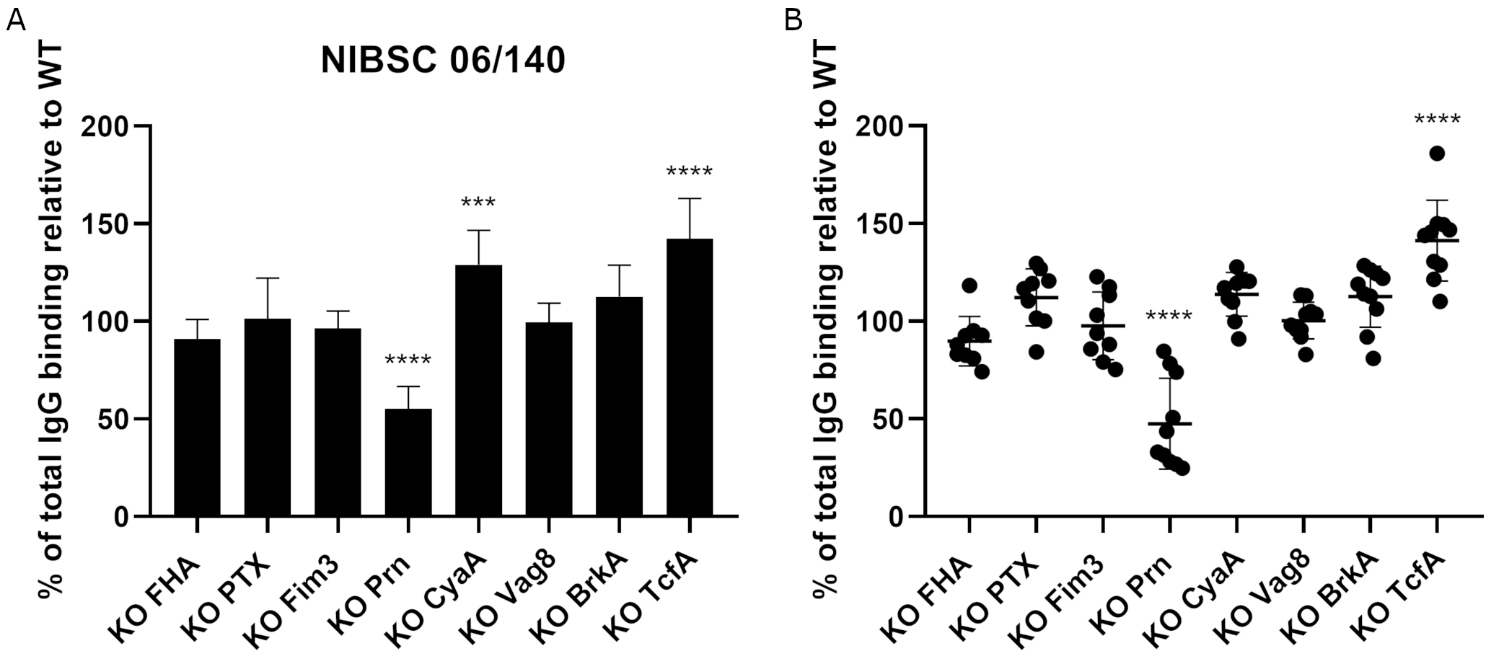
**

**Supplementary Figure 2.** **Determination of antibodies binding onto B1917 (WT) and knock-out variants using convalescent samples.** A and B. Quantification of total IgG binding onto B1917 (WT) and KO variants using the 1^st^ WHO International Standard pertussis antiserum NIBSC 06/140 (A) and 10 convalescent (B) samples. The fluorescence intensity was determined from at least two experiments with technical duplicates for panel A and at least once for panel B and reported relative to those of the WT strain. Geometric means with 95% confidence interval are represented. Statistical analyses were performed, and significant p values relative to WT strain are indicated (**** for p ≤ 0.0001, *** for p ≤ 0.001).

**
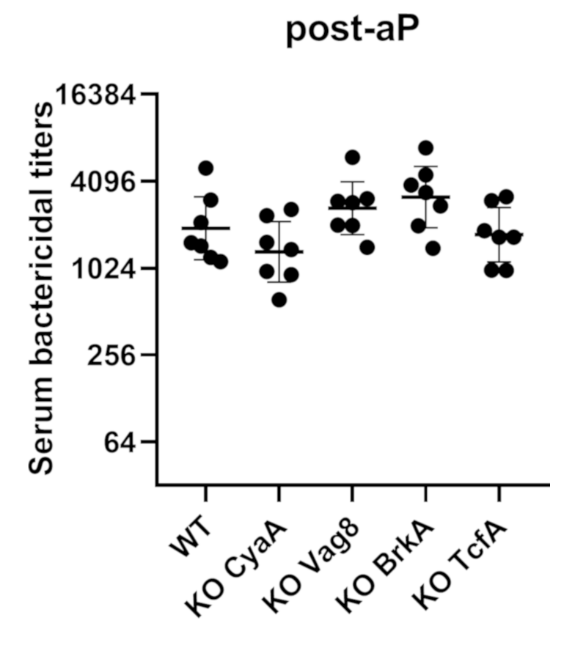
**

**Supplementary Figure 3.** **Serum bactericidal activity of post-aP vaccine samples with B1917 (WT) and KO variants deficient of vaccine candidate antigens.** Serum bactericidal titers were assigned as the interpolated serum dilution which gives 50% of bacterial killing when incubating the bacteria with serial dilutions of post-aP vaccine serum and active complement. 7 sera were analyzed. The geometric means with 95% confidence interval are represented. Statistical analyses were performed for each data point using the corresponding WT data point as a control, and no significant p values were determined.
